# Patient and Healthcare Provider Barriers to Hypertension Awareness, Treatment and Follow Up: A Systematic Review and Meta-Analysis of Qualitative and Quantitative Studies

**DOI:** 10.1371/journal.pone.0084238

**Published:** 2014-01-15

**Authors:** Rasha Khatib, Jon-David Schwalm, Salim Yusuf, R. Brian Haynes, Martin McKee, Maheer Khan, Robby Nieuwlaat

**Affiliations:** 1 Population Health Research Institute, Hamilton Health Sciences and McMaster University, Hamilton, Ontario, Canada; 2 Clinical Epidemiology and Biostatistics, McMaster University, Hamilton, Ontario, Canada; 3 Department of Medicine, McMaster University, Hamilton, Ontario, Canada; 4 Department of Health Services Research and Policy, London School of Hygiene and Tropical Medicine, London, United Kingdom; University of Tolima, Colombia

## Abstract

**Background:**

Although the importance of detecting, treating, and controlling hypertension has been recognized for decades, the majority of patients with hypertension remain uncontrolled. The path from evidence to practice contains many potential barriers, but their role has not been reviewed systematically. This review aimed to synthesize and identify important barriers to hypertension control as reported by patients and healthcare providers.

**Methods:**

Electronic databases MEDLINE, EMBASE and Global Health were searched systematically up to February 2013. Two reviewers independently selected eligible studies. Two reviewers categorized barriers based on a theoretical framework of behavior change. The theoretical framework suggests that a change in behavior requires a strong commitment to change [intention], the necessary skills and abilities to adopt the behavior [capability], and an absence of health system and support constraints.

**Findings:**

Twenty-five qualitative studies and 44 quantitative studies met the inclusion criteria. In qualitative studies, health system barriers were most commonly discussed in studies of patients and health care providers. Quantitative studies identified disagreement with clinical recommendations as the most common barrier among health care providers. Quantitative studies of patients yielded different results: lack of knowledge was the most common barrier to hypertension awareness. Stress, anxiety and depression were most commonly reported as barriers that hindered or delayed adoption of a healthier lifestyle. In terms of hypertension treatment adherence, patients mostly reported forgetting to take their medication. Finally, priority setting barriers were most commonly reported by patients in terms of following up with their health care providers.

**Conclusions:**

This review identified a wide range of barriers facing patients and health care providers pursuing hypertension control, indicating the need for targeted multi-faceted interventions. More methodologically rigorous studies that encompass the range of barriers and that include low- and middle-income countries are required in order to inform policies to improve hypertension control.

## Introduction

### Rationale

Hypertension (HT) is the leading global risk factor for mortality worldwide, responsible for 13% of deaths globally [1]. However, HT detection, awareness, treatment and control are low worldwide [2] Hypertension control at the population level involves several steps. First, those at risk must be identified [awareness]. Second, HT patients must be treated appropriately, whether with medication, lifestyle changes, or their combination. Third, they must be followed up to ensure that they are adhering to treatment and their blood pressure is controlled [3]. These recommendations are based on established research evidence yet their implementation in practice is suboptimal. Implementation can fail because of an inability to surmount barriers that relate to the patient, the health care provider, or the health system [4], [5]. Each of these has been subject to previous research but, to our knowledge, their role, importance, and generalizability has not been examined systematically thus far.

Barriers can be assessed indirectly by analyzing associations between characteristics such as region, socio-economic status, age or sex, or directly by asking stakeholders such as patients and providers about barriers they face. Indirect analysis of associations often involves non-modifiable characteristics and does not elucidate the actual reasons for subgroup disparities. Therefore we seek to address the gap in the literature by providing a systematic literature review of HT barriers as reported by patients and health care providers. Specifically, we go beyond much previous research that focused on patient adherence as the major barrier to blood pressure control. There is a need for a more nuanced approach to understanding HT control, taking account of complex interactions at different levels of care and the roles of different stakeholders involved [6]. The conceptual frameworks used in this work have been limited in scope and are often not linked to theories that might explain processes of behavior change designed to achieve optimal implementation and thereby HT control.

### Objectives

**Figure 1:**
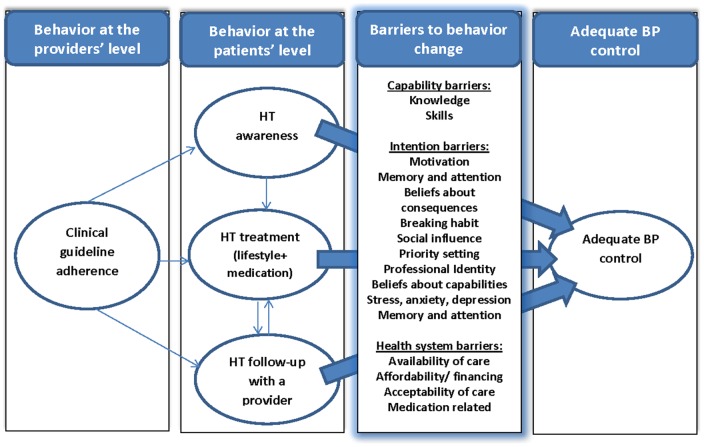
Barriers to hypertension management, modified from Michie et al (2004) and Fishbein et al (2000).

The aim of this paper is to systematically review the literature on barriers reported by HT patients as well as population groups at risk for HT [together referred to as patients from here on] and health care providers [referred to as providers from here on] that may impede optimal HT awareness, treatment, or follow up with a health care provider (*Figure 1*). This review focuses on individual level barriers, whereby barriers related to the health system are addressed only as they are reported by individuals, whether health care providers or patients. We included qualitative data to gain a better understanding of which barriers are perceived to be important from the patients' and providers' perspective, and quantitative data to assess their prevalence and their clinical importance.

## Methods

### Protocol and registration

Methods of the systematic review were specified in advance and documented in a published protocol in the International Prospective Register of Systematic Reviews (PROSPERO), registration number CRD42011001617.

### Behavior change theoretical framework

#### Definition of barriers

Barriers to HT control in this systematic review were defined as any factor limiting the performance of a required behavior by patients or providers [6] to achieve recommended HT awareness, treatment (medication and/or lifestyle) or follow up care. As indicated, non-modifiable attributes such as age, race, and gender were not considered. In keeping with best practice, we begin with a theoretical framework that encapsulates the barriers and makes it possible to explore mediating pathways and moderators [7]. The framework draws on theories from implementation research [8] and behavior change [9]. Michie (2004) proposes 12 subthemes for investigating the implementation of evidence based practice (*Figure 1*), organized under 3 main themes whereby a change in behavior requires a strong commitment for change (intention barriers), the necessary skills and abilities to perform the behavior [capability barriers], and no health system constraints [9]. These frameworks were used to organize patient and provider reported barriers, which where adapted to the specifics of HT control following an initial scoping review of qualitative studies. Barriers related to the health system are addressed in this paper only as they pertain to individuals, whether health care providers or patients.

#### Definition of themes

**Capability barriers** may relate to the knowledge of behaviors required to achieve HT control, or the capacity to perform these behaviors. **Intention barriers** relate to attitudes or motivations towards actions necessary to achieve control and may be mediated by several behavioral characteristics. **Health care system barriers** may include barriers that are external to patients' or health care providers' control [10]. These include availability of resources [inputs], financing and affordability, and the mode of delivery and acceptability of health services. These barriers also extend beyond the healthcare system to the wider health environment, and include other facilities required for a healthier lifestyle. In addition, medication related barriers for patients, such as side effects, were included under health system barriers as they are also out of patients' control (*Figure 1*).

### Information sources and search

Studies were identified by searching electronic databases, scanning reference lists of included articles and consultations with experts in the field. No limits were applied with respect to language and those in languages other than English were translated. The search was applied to MEDLINE (1948 to January, 2013), EMBASE (1980 to 2013 Week 09) and Global Health (1973 to January 2013). An experienced librarian helped in developing the search strategy to identify studies (*table S1 in file S1*). Controlled vocabulary and keywords focused on “hypertension”, “barriers”, and “obstacles”. No limits to study design were imposed.

### Eligibility criteria and study selection

Table 1 shows the eligibility criteria for studies. Two reviewers independently assessed studies identified by the search for eligibility based on the title and abstract. Selected full text papers were then assessed independently by the two reviewers using a standardized form that was piloted on 6 studies designed to describe the characteristics of studies to be included based on recommendations in the Cochrane Handbook section 5.1.0 [11]. Disagreement was resolved by a third author. Unweighted kappa for the second screening phase was calculated using PC-AGREE software (version 2.5) to assess agreement between the 2 reviewers and revealed an excellent agreement beyond chance of 0.87 (±0.09) [11].

**Table 1:** Eligibility criteria.

*Types of participants*:
Patient populations of any age, with a HT diagnosis or at risk for HT.
Health care provider populations were considered without restrictions to the type of health care provider [physician, nurse, other], level of practice [primary care vs. hospital level], or the population they cater to.
*Study outcome/focus*
**HT awareness**; detection, screening.
**HT treatment**: Medication intake, medication adherence, clinic visits
**Lifestyle change**: diet, physical activity, alcohol intake, weight loss
**Follow up** with a health care provider for HT management
**Clinical guideline** adherence, medication prescription
Studies that focused on BP control in general, without specifying an outcome leading to control as specified above were excluded.
*Types of studies*:
Qualitative and quantitative observational studies assessing barriers to HT awareness, treatment (medication and lifestyle), or follow-up care. Effectiveness (RCT) and comparison (cohort, case-control) studies were included only if a barrier assessment was assessed within the study.
Studies were included regardless of study quality
No language or publication date restrictions were imposed.
Conference abstracts and non- peer review studies were excluded.

### Data collection and data items

Two reviewers independently extracted data from included studies using a form that was piloted on 4 randomly included studies. The following information was extracted from each study: Study characteristics [qualitative or quantitative design, overall objective, setting, participant characteristics], barriers assessed in the study, information on use of theory or validated tools to assess barriers, prevalence of reported barriers, outcome measures (effect of barrier on adherence to medication) when available.

### Study quality assessment

Risk of bias was assessed at the study level. Following the Cochrane Collaboration's recommendation to present potential biases for each study instead of using scores to rate quality, a set of quality appraisal items relevant to the type of studies included was applied (tables S3 and S4 in file S1). Quality of included qualitative studies was assessed using an existing framework and its set of validated tools [12]. This framework was selected for this review due to its applicability among the different types of included studies and ease of presentation. For quantitative studies, these included biases in sample selection, quantification of barriers, measure of the outcome, and appropriateness of statistical analysis i.e adjusting for confounders when applicable.

### Data synthesis and analysis

Studies were classified as qualitative or quantitative based on authors' description, and were organized according to the theoretical framework separately for patients and providers. Qualitative data investigates why and how certain barriers affect the outcome of interest [13]. Consequently we used these data to modify and explain themes according to the framework. We then used quantitative data to quantify how common these barriers were. Classification of barriers into the framework's subthemes was done independently by two reviewers; discrepancies were resolved by a third reviewer.

#### Qualitative data analysis

Results from qualitative studies were analyzed using descriptive analysis reporting in how many studies barriers of the framework domains arose, with specific examples of barriers to clarify the domains.

#### Quantitative analysis

Once barriers from each quantitative study were organized into the framework, the proportion of participants reporting each barrier was extracted [when reported]. This generated a measure of how frequently each barrier was reported and facilitated identification of barriers that might be inadequately studied in the literature, in the same way that was done in a previous study of barriers and facilitators of adherence to treatment for highly active antiretroviral therapy (HAART) [14], showing the number of studies assessing each barrier. The extracted proportions were then pooled in order to identify how prevalent these barriers were across the different study populations included in this review. When the same study had more than one question or statement assessing the same barrier, the median prevalence was calculated. This was done in order to prevent pooling of duplicate results from the same study, which would result in an overestimation of the pooled proportion [15].

The inverse variance method was used to pool proportions presented in each study. Review manager 5 was utilized to conduct these calculations. The proportion of study participants reporting the barrier (p) and the study sample size were used to calculate the standard error (SE(p)), using the following formula: SE (p) = sqrt ((p)(1-p)/n [12].

### Summary measures and additional analyses

Association measures for barriers with the outcome of interest were also pooled and stratified by the frameworks subthemes. Four of the five studies that provided effect measures used odds ratios, the remaining study used hazard ratios [16]. Risk was assumed similar for these two measures and they were pooled together, sensitivity analysis was conducted by excluding the study reporting hazard ratios. Only adjusted effect measures were pooled.

Due to expected heterogeneity in the included studies the random effects model was used to pool the data, making an adjustment to the study weights according to the extent of variation of proportions from each study. Using a random effects model does not explain or justify heterogeneity, yet it provides wider confidence intervals [11]. Pooled proportions and pooled effect measures are presented using forest plots depicting the 95% confidence interval, the I^2^ statistic, and the number of pooled studies. The I^2^ statistic describes the percentage of the variability in effect estimates that is due to heterogeneity rather than sampling error (chance) [11].

## Results

### Study selection

**Figure 2:**
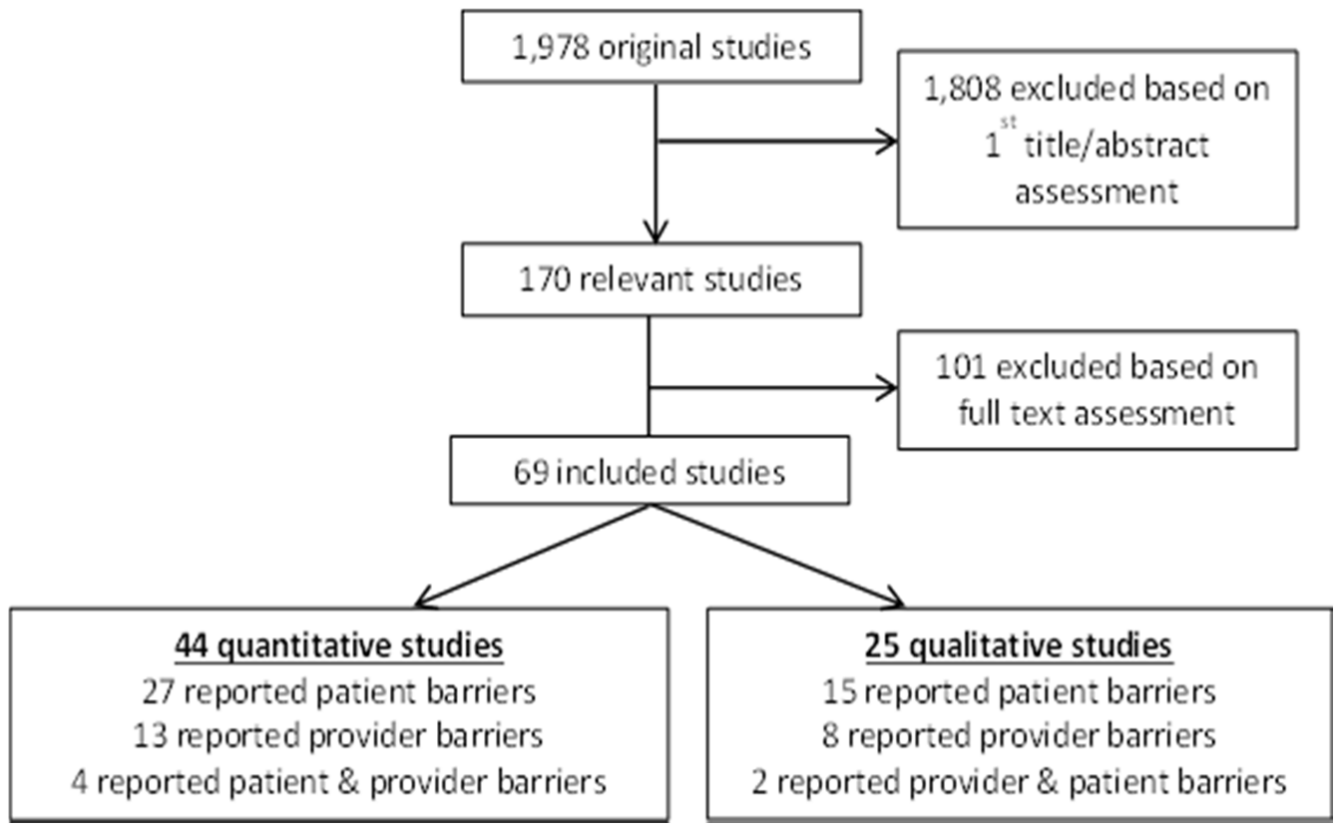
Flow diagram of included studies.

The search identified a total of 1,978 articles (*Figure 2*). Of these, 1,808 articles were excluded in the 1^st^ screening based on title/abstract reviews. The full texts of the remaining 170 citations were examined in more detail in the 2^nd^ screening, of which 69 studies (25 qualitative, 44 quantitative) were included in the review. Three included studies were translated from Russian [17], Portuguese [18], and Korean [19] into English.

### Study characteristics

Eight qualitative and 13 quantitative studies reported provider barriers. Fifteen qualitative and 27 quantitative studies reported patient barriers. Two qualitative and 4 quantitative studies reported both patient and provider barriers. *Table 2* presents a summary of study characteristics. The majority of studies were conducted in high income countries (HIC), mainly in the USA, with only 14 (20%) in lower or middle income countries (LMIC). Among patient studies, 28% were population based, while the remaining studies recruited patients from clinic or hospital settings. Among providers, 33% (n = 7) included non-physician health care workers in their sample (nurses, pharmacists, social workers…) (*tables S2 and S3* in file S1).

**Table 2:** Study characteristics (n = 69).

	Number of studies		
	Qualitative	Quantitative	Total
**High income countries**			
USA	15	22	**37**
UK	2	2	**4**
Canada	0	3	**3**
Other^1^	5	6	**11**
**Middle and lower income countries** 2	3	11	**14**
**Study setting**			
Primary/secondary care	19	35	**54**
Community	6	9	**15**
**Study type**			
Focus groups	16	NA	**16**
In depth interviews	7	NA	**7**
Focus groups and interviews	2	NA	**2**
Cross sectional	NA	41	**41**
RCT baseline	NA	1	**1**
RCT follow up	NA	2	**2**
**Study population**			
Only hypertensive patients	12	26	**38**
Other chronic disease patient or general community	5	5	**10**
Physicians only	3	11	**21**
Other health care workers [nurses, pharmacists…]	5	2	
**TOTAL**	**25**	**44**	**69**

^1^ Australia, Republic of Korea, Israel, Netherlands, Kuwait, Switzerland, Ireland, Singapore, Europe, Croatia.

^2^ qualitative studies: India, South Africa, Brazil, Malaysia, Nigeria, Trinidad & Tobago, China, and Russian Federation.

NA = No studies Available.

### Study quality

Risk of bias for each study is presented in tables S4 and S5 in file S1. For **qualitative studies**, only 1 of the 25 studies explicitly assessed the likely impact of the authors own personal characteristics on the data obtained (reflexivity). The context or setting of the study was inadequately described in 40% (n = 10) of studies, and 68% (n = 17) of studies failed to support their methods or results by a theoretical framework or a wider body of knowledge. As for **quantitative studies** 84% (n = 37) reported a response rate lower than 85%, and 68% (n = 30) did not use a validated tool/instrument to assess barriers.

## Results of Individual Studies and Synthesis of Results

*Table S6* in File S1 presents the number of qualitative studies in which barriers were reported, according to the framework.

### Provider reported barriers in qualitative studies

#### Capability barriers

**Knowledge** barriers were discussed in two studies that were conducted by the same group [20], [21]; providers reported that lack of knowledge regarding HT management was not a barrier to HT control but there were reports of unfamiliarity in how best to manage certain subgroups like the elderly with comorbidities. **Skills** barriers mainly included difficulty in keeping up with new clinical information [20], educating and counselling patients [22] and addressing prehypertension [23].

#### Intention and determinants of intention strength

**Motivation** barriers pertained to the intention to perform the action. Providers reported the difficulties and repeated failures in addressing healthy behaviors and achieving a controlled blood pressure resulting in lack of motivation to try [22]–[24]. **Beliefs about consequences** related to concerns about medications, clinical guidelines, and other recommendations. Providers doubted the efficacy of certain medications [25] or were reluctant to initiate aggressive anti-hypertensive drug treatment due to possible side effects [24]. Some providers doubted whether following clinical guidelines would improve outcomes [26]. Providers raised concerns about the accuracy and representativeness of individual BP readings during the visit as well as concerns regarding white coat effect when taking these readings [20], [21]. **Breaking habit** was another barrier, where providers reported satisfaction with their current performance [26], suggesting reluctance to change their habits or routines [clinical inertia].

**Social influence** barriers included lack of care coordination with colleagues as well as social pressure and conflicting roles in a practice. Providers described their reluctance to initiate treatment for ‘someone else's patient’ despite repeated recording of high BPs [21]. Poor coordination between different general practices and lack of consensus in standardization of measurements were also reported [20], [23], [27]. Problems with **Priority setting** may sometimes prevent better HT control. For example, other acute medical conditions competed for attention with HT during the visit [26], [27] making it harder to prioritize HT care. **Professional identity** was commonly discussed in terms of lack of trust in the evidence on which guidelines were based upon [21], [26]. Providers also reported that guidelines may not always be practical and do not necessarily translate to everyday [24]. One study invoked **beliefs about capabilities**, suggesting that providers cannot perform according to the guidelines [26]. **Emotional** barriers, which may include issues relating to stress or burn-out due to high workloads, or to anxiety/depression, and **Memory and attention** barriers were not reported by providers.

Health systems related barriers. Health system barriers were the most commonly reported barriers among providers. Those relating to **Availability** of health care resources included lack of consultation time [22], [23] which may impair the ability to follow guidelines, resulting in poor BP control. Lack of space, equipment, and shortage of staff were also reported [22]. In atypical settings, disruption of treatment due to severed supply channels and inoperable pharmacies following disasters were also reported [28]. Providers also reported difficulties in locating guidance on delivering care [21], [22]. **Affordability** barriers included insufficient financial reimbursement or incentives to apply recommended HT care [21], [23], [24], [26], [27]. None of the providers reported any barriers regarding providers' **acceptability** of health care or medications.

It is important to note that provider-focused studies also reported the patient to be a barrier to guideline adherence; for example providers stated that patients were reluctant to take more medications [21] and they wanted to try changing their lifestyle before starting drug therapy [21], thus creating a barrier for providers seeking to follow clinical guidelines. Providers also reported patients' resistance to change to a healthier lifestyle, as well as patient stress and comorbidities [26] as a barrier to BP control. Since these barriers were patient-specific and are external to providers they were not coded under provider barriers.

### Patient reported barriers in qualitative studies

#### Capability barriers

**Knowledge** of HT risk factors varied by study and within study; some participants were aware that a poor diet, high salt and fat intake, and lack of physical activity might be a risk factor for HT [29], whereas others reported less knowledge of such risk factors [25], [29]. Smoking and alcohol were reported as risk factors in one study only [18]. Patients were not familiar with blood pressure readings and their meaning [30]. Gaps in understanding risk factors to and consequences of HT were reported [18], [25], [29]–[32]. Patients reported the need for better education regarding HT management and prevention [29], [30], [33], and suggested that, in comparison with HT, they receive more information regarding diabetes [34]. In one study, not knowing about the existence of screening service was reported as a barrier to awareness [35]. **Skills** were discussed in terms of communication between patients and providers, such as not feeling guilty about asking questions and knowing what questions to ask [36]. Lack of skills to check blood pressure at home were also discussed [30].

#### Intention and determinants of intention strength

**Motivation** barriers refer to intention to change and were reported in terms of exercise, where patients described being too lazy or too tired to exercise [37]. Lack of motivation was also reported in terms of medication adherence, where patients admitted to not putting enough effort or thought to taking their medication as prescribed [34]. **Beliefs about consequences** of taking medication were commonly discussed; participants believed that they did not need anti-hypertensive medication because they have no symptoms [36], [38], they denied the diagnosis and viewed it as a reaction to stressful events and not necessarily a chronic disease [34]. Patients also expressed fear of “dependence” on anti-HT medications if they continue to take them [36], [39] and preferred modifying their lifestyle over taking medication [40]. Beliefs about the consequences of a healthy lifestyle were also discussed [31], African American patients, for example, were reported as considering HT as being inevitable [41], similarly some patients showed a fatalistic perspective suggesting that “it's all in God's hands” [33]. Therefore improving diet or exercising might not make any difference. **Breaking habit** barriers were mostly reported in terms of adapting to a healthier lifestyle, whereby patients mainly expressed difficulty in changing dietary habits [22], [33]. Difficulties with making long term medication adherence a habit were also identified [25], [36], [37].

**Social influence** was reported as both a barrier and a facilitator of improved HT control. Lack of social support, mainly from the family, affected medication adherence [25], [36] and changing lifestyle [37]. Studies also reported that having to cook for oneself differently from the rest of the family was perceived as a barrier [22], [31], [37], [41]. In terms of utilizing health care serves and screening for HT, participants suggested that sessions aimed at increasing health awareness should include groups of patients and be social [35]. Social pressure was also reported as a barrier to a healthier lifestyle [33], [40]. **Prioritizing** one's health was also reported as a barrier. Participants found it hard to prioritize clinic visits, diet and exercise over needs of family members [22], [34], [38], [40]–[42] and over work [35], [37], [42]. Patients reported that **stress and anxiety** may affect HT management; such emotions maybe related to lack of money and jobs, single parenting, and living in unsafe neighbourhoods [31], [34], [37], [39], [41]. **Memory** or forgetting to take one's medication appeared to play an important role in medication adherence [30], [36]. **Beliefs about capabilities** were not discussed in any of the included studies.

#### Healthcare system barriers

**Availability** barriers were relevant to lifestyle change as well as medical treatment. Patients reported lack of facilities, bad weather, and safety issues as barriers to physical exercise [33], [37]. Barriers to following a healthy diet included absence of nearby stores that sell healthy foods [39], limited healthy food choices when eating out [22], and lack of guidance and dietary counselling from clinicians [30]. In terms of utilizing care, patients reported difficulties with transportation [29], inappropriate hours for screening services that conflict with working hours [35], and difficulties in getting clinic appointments [36], or absence of or inaccessible health care facilities [25], [33]. Other availability barriers included transportation difficulties hindering medication refills [30], [42], no interpreter services in physician offices [29], lack of information targeting population subgroups such as African Americans [41], or short duration of physician consultations [25].

**Affordability** of care barriers included lack of insurance and high costs of treatment [30], [38], [40] resulting in patients seeking care only for acute problems [25], [28], [29], [37], [41], [42]. Cost issues also limited the ability to follow a healthy diet [31], [37], [41] and to exercise [33]. **Acceptability** of available care included poor provider-patient communications [36], patients' distrust in the services provided [33], [42], lack of respect for the poor [25], and lack of attention to minorities [30], [42]. **Medication** related barriers mainly included side effects experienced due to anti-HT medications [34], [36]–[39], as well as dosing frequency, taste, and large pill size [36].

### Provider reported barriers in quantitative studies

**Figure 3:**
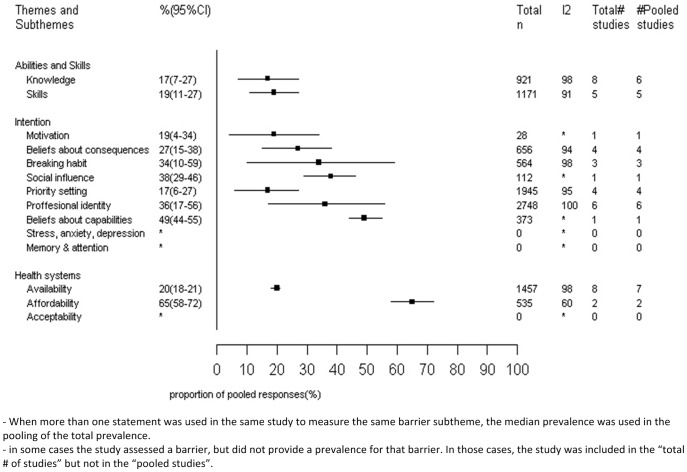
Pooled prevalence (%) and 95% confidence intervals (CI) of provider level barriers to hypertension management organized by Michie et al framework (n = 13).

Figure 3 presents the pooled prevelence of barriers reported by providers from 13 studies [32], [43]–[54]. In terms of **capability** barriers, 19% (95%CI: 11–27%) of providers reported that their lack of skills contributed to suboptimal BP control. 17% (95%CI: 7–27%) reported either directly or indirectly (by means of some measure of their knowledge) lack of knowledge as a barrier. Belief that one's capabilities to manage and control HT were limited was the most common of **Intention** barriers (49% of providers), though it was only assessed in one study. This was followed by social influence from peer providers (38%, 95%IC: 29–46%) and providers' disagreement with guidelines (36%, 95%IC: 17–56%). In terms of **health system** barriers, low salaries and lack of reimbursements were most often reported as barriers among providers (65%, 95CI: 58–72%).

### Patient reported barriers in quantitative studies

**Figure 4:**
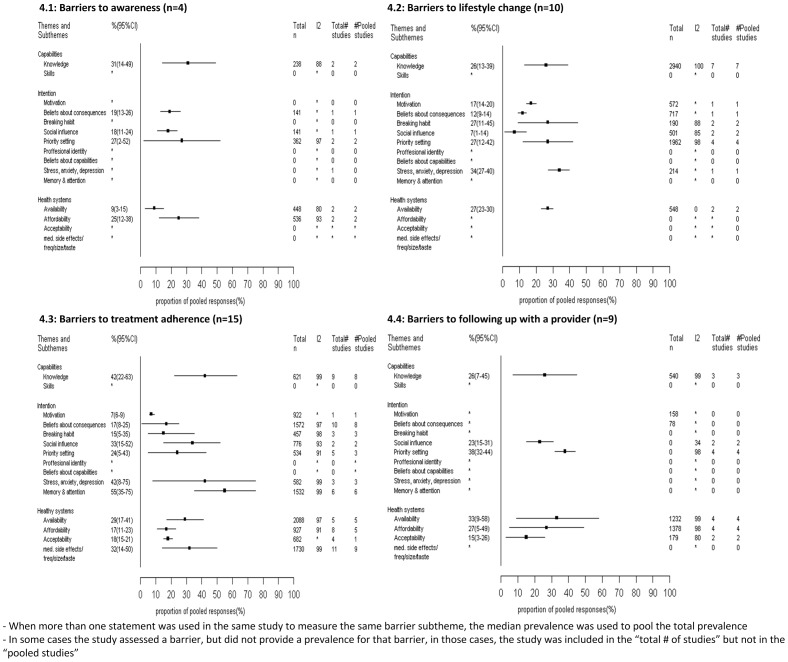
Pooled prevalence (%) and 95%CI of patient level barriers to hypertension management organized by Michie et al framework (n = 32).

*Figure 4* presents the pooled prevalence of barriers to each HT management outcome organized by subthemes of the framework. Four studies reported barriers to hypertension awareness (figure 4.1) [55]–[58], 10 studies reported barriers to lifestyle change (figure 4.2) [44], [45], [59]–[66], 15 studies reported barriers to treatment adherence (figure 4.3) [16], [43], [58]–[60], [67]–[76], and 9 studies reported barriers to following up with a health care provider (figure 4.4) [44], [58], [59], [62], [65], [73], [77]–[79].

Of the 2 **capability** subthemes, only knowledge was assessed, and was mostly reported as a barrier to HT medication treatment adherence, reported as a barrier by 46% (95%CI:24–64%) of patients. In terms of **Intention barriers**, memory and attention barriers were of most importance to patients in terms of medication adherence (55%, 95%CI:35–75%). In terms of changing lifestyle, stress/anxiety was mostly reported (34%, 95%CI: 27–40%), but results were based on one study only. Priority setting (27%, 95%CI: 12–42%) and breaking habit (27%, 95%CI: 9–45%) were more commonly assessed and also appeared to be prevalent barriers to lifestyle change. Priority setting was again the most commonly reported barrier to HT screening and follow up with a provider (38%, 95%CI:32–44%). As for **health care system barriers**, availability (29%, 95%CI:17–41) of medication and its side effects (29%, 95%CI: 9–49%) were the most common barriers to patients' medication treatment adherence and persistence. For seeking HT screening, affordability barriers (28%, 95%CI: 2–53%) were more commonly reported than availability barriers. And finally in terms of following up with a provider, availability barriers had the highest prevalence (33%, 95%CI:9–58%).

### Clinical importance of barriers

**Figure 5:**
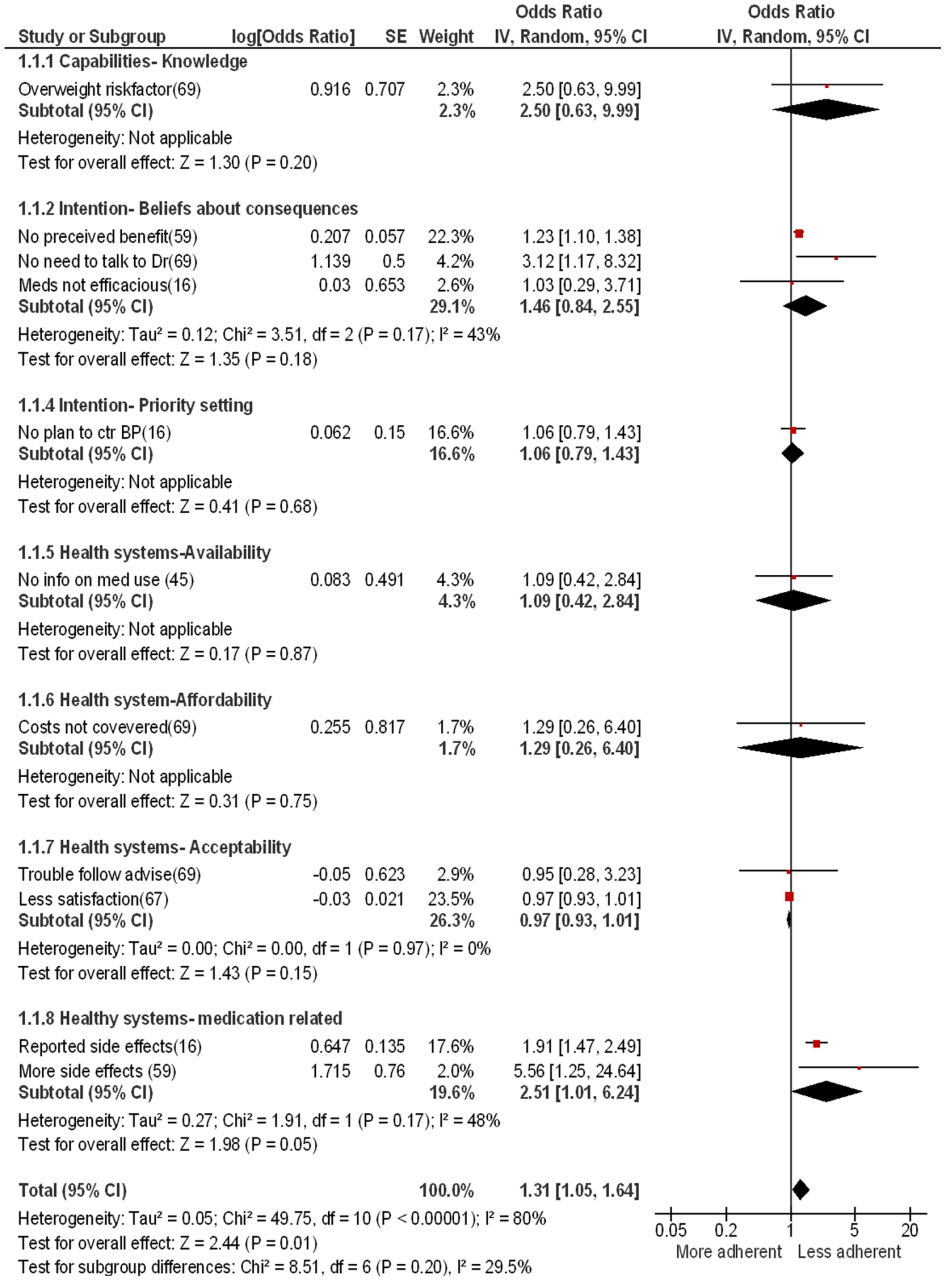
Pooled effect of barriers on hypertension treatment adherence/persistence (n = 5).

None of the provider studies reported measures of association of barriers with guideline adherence. Therefore, it is not possible to assess to what extent the provider reported barriers were actually associated with worse care. For patients, it was possible to assess the association of barriers with HT treatment adherence based on 5 studies that provided an adjusted effect measure [16], [59], [66], [67], [69], [74]. Figure 5 shows that overall reporting of at least one barrier was associated with an increased risk of non-adherence (OR: 1.27, 95%CI: 1.00–1.58). Heterogeneity was very high (I^2^ = 78%), and excluding the one study that reported hazard ratios instead of odds ratios did not explain heterogeneity (OR = 1.28, 95%CI: 1.03–1.60), 1^2^ = 80%.

Stratifying the barriers by subthemes of our framework explained most of this heterogeneity. Only one study reported a measure for **capability** barriers, suggesting a non-statistically increased risk of non-adherence among those with lower HT knowledge. Data was available on only 2 of the **intention** subthemes and suggested a non-statistically significant trend towards higher non-adherence among patients reporting barriers. Finally, all 4 **health systems** subthemes were assessed in terms of their effect on non-adherence, 3 of which [availability, affordability, acceptability] indicated a non-statistically significant trend towards higher non-adherence among patients reporting barriers. Patients reporting medication side effects had a statistically significant two fold increased risk of non-adherence (OR: 1.92, 95%CI: 1.47–2.49, I^2^ = 0%).

### Comparison between health system barriers in HIC and LMIC

#### Provider barriers

Only 3 studies reported qualitative data from LMIC [India [25], Brazil [80], and South Africa [22]]. Differences with HIC appeared mainly in terms of availability barriers; providers in LMIC reported shortages of space, equipment and staff [22], [25]. These barriers were not reported in HIC. These differences were also observed in data from the 4 quantitative studies conducted in LMIC (Nigeria [45], Russian federation [17], Trinidad [48] and China [52]). These studies were more likely to assess and report lack of equipment, medication, time [48], and specialists [17]. The studies from HIC focused more on issues relating to availability of guidelines [46] and organization of follow up care [43].

#### Patient barriers

Qualitative studies of patients in HIC focused on lack of exercise facilities and healthy food choices, while patients in LMIC on the other hand were more likely to report lack of health care facilities [25]. In terms of acceptability, LMIC [25] reported barriers similar to those reported by ethnic minorities in HIC [30], [41].

Among quantitative studies that provided enough data to pool the prevalence of patient barriers, only seven were from LMIC; 2 from South Africa [64], [68], and one from each of Malaysia [67], Egypt [59], Singapore [81], Trinidad [60], and India [76]. Only one study assessed barriers to screening [69], two studies assessed barriers to medical adherence [64], [81], and two assessed barriers to following up with a health care provider [59], [81].

## Discussion

### Summary of evidence

Among qualitative studies, health system barriers, specifically availability barriers, were most commonly discussed as barriers to HT management for patients and providers. For providers, availability barriers included lack of resources and time, and a high workload. For patients, availability barriers were related to distance and transportation to primary health care centers and pharmacies, as well as proximity of physical activity facilities and grocery stores that sell fresh fruits and vegetables. This was different from quantitative studies, where researchers focused on barriers related to knowledge and professional identity/agreement with guidelines among providers. Among patients, researchers focused on beliefs about treatment consequences, and side effects of medications.

The prevalence of the various barriers in quantitative studies varied; this reflects the heterogeneity of study populations and methodologies of the quantitative studies and, in particular, the extent to which barriers were sought. However, with these caveats, it was possible to make some inferences on which barriers were most prevalent in terms of HT management. Affordability barriers included insufficient financial reimbursement or incentives to apply recommended HT care were most common among providers.

For patients, very few studies assessed barriers to awareness [screening], likely because such studies require general population studies which are more difficult to mount than clinic based studies of HT populations. Knowledge barriers regarding the importance of HT and BP screening, appear to be the most common barriers to HT detection [awareness]. Stress, anxiety and depression barriers were most commonly reported in terms of lifestyle change. In terms of patient persistence and adherence with HT treatments, patients mainly reported forgetting to take their medication or were unsure if they had already taken their medication. Finally, priority setting for regularly scheduling visits to their healthcare provider was often reported by patients.

Our review suggests that knowledge barriers were commonly assessed, yet they were not always the most prevalent barrier. Similar observations can be made about intervention studies to improve BP control; a Cochrane review identified 72 clinical trials, of which 30 assessed education interventions directed either at patients or providers but they were not effective at improving BP control [57]. The same review reported that self-monitoring and appointment reminders may be useful but require further evaluation [82]. These programs likely affect intention barriers which, based on our review, require further study. Understanding these barriers may help develop more effective interventions for improving blood pressure control.

Previous reviews have identified possible barriers to HT management [6], [83], yet none have done so systematically. These reviews acknowledge that different factors affect HT control, whether patient related or provider related. Our review systematically reviewed the literature and suggests that barriers are likely different for different stakeholders, settings and outcomes. Nevertheless, some commonalities across settings were found, which should become more meaningful with standardized barrier assessment methods.

Knowledge translation models suggest that success is more likely if strategies are informed by and tailored to an assessment of possible barriers and facilitators [84]. This review provides a framework to help in this process. The framework also offers a means for future researchers to present their results in ways that provide greater conceptual clarity on the nature of interventions, increasing the chances of designing more effective implementation interventions and translating evidence into improved HT control [85].

### Limitations

The methodological quality of both qualitative and quantitative studies was modest. Surveys were rarely validated and their development was usually not explicitly based on theory or previous qualitative analyses. Other reviews in the literature of barriers to medication adherence support these findings [86]. Further, studies mainly focused on providing prevalence of reported barriers and very few studies measured how these barriers actually might affect HT control by assessing measures of association. The majority of included studies were conducted in higher income countries, mainly the USA, and thus results may not necessarily be applicable to other high income countries and lower and middle income countries. Though the literature acknowledges that poor HT control is determined not only by patient barriers but also provider barriers [6], the larger number of patient studies included in this review indicates that research is still focused on assessing barriers at the patient level, rather looking at other stakeholders. Further, included studies focused on treatment and control of HT, whereby intention barriers seemed understudied, and very few assessed barriers to HT awareness.

The I^2^ statistic was high even though pooled proportions were stratified by study outcome [awareness, lifestyle change, treatment adherence and following up with providers]. Studies were heterogeneous in terms of the study population, study setting, use of theory, and barrier assessment methods and tools. We pooled prevalence of each barrier primarily for illustration, and the pooled results should therefore be interpreted with caution. Considerable heterogeneity has been observed in previous studies pooling proportions of barriers reflecting the nature of the underlying research [14]. An additional issue is the need to understand better the role of context-specific factors relating to the population and the setting being assessed.

A more systematic way of measuring these barriers, using standardized and validated methods, is necessary. Very few studies actually assessed the three main themes of the proposed theoretical framework, and none incorporated aspects from all 12 subthemes. Using a theoretical framework to measure all the barriers, with the same methodology, might provide a more reliable way to compare the prevalence and clinical importance of these barriers between different settings.

### Implications

To improve HT control, intervention should overcome capability barriers, intention barriers, and health system barriers. These barriers should be targeted at the provider and the patient level. More methodologically rigorous studies that consider all the different barriers and that include lower income countries are required in order to improve our confidence in determining the most important modifiable barriers, to compare them among regions and populations, and to develop interventions tailored to different settings and types of patients to improve HT control.

## Supporting Information

Checklist S1 **PRISMA checklist.**
(DOC)

File S1 **Supporting tables.** Table S1, Search strategy (Medline). Table S2, Detailed study characteristics of included qualitative studies. Table S3, Detailed study characteristics of included quantitative studies. Table S4, Quality appraisal of qualitative studies. Table S5, Quality appraisal of quantitative studies. Table S6, Counts and examples of barriers per theme among qualitative studies.
(DOCX)
