# Supplementary material for: Patient and Healthcare Provider Barriers to Hypertension Awareness, Treatment and Follow Up: A Systematic Review and Meta-Analysis of Qualitative and Quantitative Studies
Source: PLoS One. 2014 Jan 15;9(1):e84238. doi: 10.1371/journal.pone.0084238 (PMC3893097; doi:10.1371/journal.pone.0084238)
Supplement: File S1 — Supporting tables. Table S1, Search strategy (Medline). Table S2, Detailed study characteristics of included qualitative studies. Table S3, Detailed study characteristics of included quantitative studies. Table S4, Quality appraisal of qualitative studies. Table S5, Quality appraisal of quantitative studies. Table S6, Counts and examples of barriers per theme among qualitative studies. (DOCX) [file pone.0084238.s002.docx]

**Supporting information:**

**Table S1: search strategy (Medline)**

|  | **Searches** | **Results** |
| --- | --- | --- |
| 1 | exp hypertension/ | 197869 |
| 2 | hypertens$.tw. | 284429 |
| 3 | exp blood pressure/ | 233640 |
| 4 | (blood pressure or bloodpressure).tw. | 197506 |
| 5 | 1 or 2 or 3 or 4 | 554665 |
| 6 | exp Guideline Adherence/ or exp Nurse Practitioners/ or exp Evidence-Based Medicine/ or exp Family Practice/ or exp Adult/ or exp Group Processes/ or facilitator*.mp. or exp Diabetes Mellitus, Type 2/ | 5259880 |
| 7 | "health care facilities, manpower, and services"/ or advance directive adherence/ or guideline adherence/ or "health care quality, access, and evaluation"/ or "delivery of health care"/ or health services research/ | 98872 |
| 8 | exp Medication Adherence/ | 3491 |
| 9 | concordance.mp. | 21649 |
| 10 | exp Health Knowledge, Attitudes, Practice/ or exp Attitude to Health/ or exp "Attitude of Health Personnel"/ | 337276 |
| 11 | exp Evidence-Based Medicine/ | 45306 |
| 12 | exp Health Education/ or exp Access to Information/ | 127977 |
| 13 | exp Health Behavior/ or exp Attitude to Health/ | 270858 |
| 14 | exp Health Promotion/ | 44829 |
| 15 | exp Health Behavior/ or exp Health Education/ | 196397 |
| 16 | exp Health Services Accessibility/ | 73251 |
| 17 | exp "Attitude of Health Personnel"/ | 107170 |
| 18 | exp Physician's Practice Patterns/ | 34408 |
| 19 | exp guideline/ or practice guideline/ | 22013 |
| 20 | exp "Delivery of Health Care"/ | 694729 |
| 21 | exp Access to Information/ | 3431 |
| 22 | exp Health Services Accessibility/ or accessibility.mp. | 92630 |
| 23 | exp Polypharmacy/ or exp Drug Combinations/ or polypill.mp. | 56142 |
| 24 | 6 or 7 or 8 or 9 or 10 or 11 or 12 or 13 or 14 or 15 or 16 or 17 or 18 or 19 or 20 or 21 or 22 or 23 | 5817930 |
| 25 | barrier*.mp. | 140834 |
| 26 | facilitator.mp. | 2757 |
| 27 | obstacle.mp. | 9666 |
| 28 | 25 or 26 or 27 | 152556 |
| 29 | 24 and 28 | 47232 |
| 30 | 5 and 29 | 1120 |
| 31 | exp Policy/ or exp Organizational Policy/ or exp Nutrition Policy/ or exp Policy Making/ or exp Health Policy/ | 118195 |
| 32 | exp Legislation, Medical/ or exp Legislation/ or exp Legislation as Topic/ or exp Legislation, Drug/ | 136128 |
| 33 | organizations/ or exp government/ or exp government agencies/ or exp health planning organizations/ or exp international agencies/ or exp organizations, nonprofit/ | 164593 |
| 34 | 31 or 32 or 33 | 364850 |
| 35 | 24 or 34 | 6033781 |
| 36 | 28 and 35 | 48556 |
| 37 | 5 and 36 | 1126 |

| Table S2: detailed study characteristics of included qualitative studies | | | | |
| --- | --- | --- | --- | --- |
| Study, year,  Participants | **Country** | **Recruitment site** | **Study focus** | **Population** |
| Anthony, 2012  Patients | Israel | Large publicly  financed health organization | to gain a deeper understanding of the beliefs, attitudes and coping mechanisms of patients with HT | Diagnosed with HT, with and without diabetes |
| Aroian, 2012  Patients | USA | Hispanic professional  organization and a food service worksite | To explore attitudes and beliefs related to prevention and control of high BP among a diverse group of Hispanics living in Orange County, central Florida. | Not specific to HT patients |
| Barnes, 2012  Patients | USA | two primary care clinics utilized by immigrants and Mexican Americans | Describe knowledge of Mexican immigrants and Mexican Americans and their experiences with a diagnosis of hypertension as they  lived with and engaged in care for high blood pressure | Diagnosed and treated for HT |
| Horowitz, 2004  Patients | USA | Harlem hospitals | Explore patients’ perceptions of their condition, and the  role of certain factors, in causing and controlling HT | Diagnosed and treated for HT |
| Fongwa, 2008  Patients | USA | Routine clinic visits | Identify factors associated with adherence to HT treatment in African American women | Diagnosed and treatment for HT |
| Ford, 2009  Patients | USA | Local churches of urban communities | To describe the perceptions of hypertensive Southern, rural African American women regarding factors that affect HT | Diagnosed with HT |
| Greer, 2010  Patients | USA | Outpatient clinic | To examine African American patient perceptions of racial discrimination in clinical encounters | Diagnosed with HT; kept 2 appointments |
| Machado, 2012  Patients | Brazil | Centre of reference in cardiovascular diseases (CRDC) | To understand perceptions of hypertensive patients regarding risk factors and experiences with high BP in the city of Salvador. | Diagnosed with HT |
| Murimi, 2010  Patients | USA | Local organizations, schools, and churches | Investigate personal, cultural, and external barriers that interfered with participating in a community preventive outreach program | Not specific to HT patients |
| Ogedegbe, 2003  Patients | USA | Two primary care practices | Perspectives of hypertensive African American patients regarding the factors they perceived as barriers or facilitators to adherence | HT patients taking at least 1antihypertensive |
| Park, 2012  Patients | Republic of Korea | local health center in Seoul | to develop an effective education program for hypertension based on health belief model | hypertensive or pre-hypertensive patients |
| Peters, 2006  Patients | USA | Schools and churches | To explore the behavioral, normative, and control beliefs of African Americans relative to initiating and maintaining self-care behaviors necessary to control BP and prevent HT | Healthy community dwellers |
| Pham, 1999  Patients | USA | Organizations,  local leaders, churches | Awareness and understanding of chronic conditions, health care barriers, and cultural beliefs in Philadelphia Vietnamese community | Not specific to HT patients |
| Schafheutle,2002  Patients | UK | Three community pharmacies | How charges for medicines incurred by patients influence their decisions for managing acute or chronic conditions | Not specific to HT patients |
| Wexler2, 2009  Patients | USA | The Ohio State University | Identify barriers and understand beliefs and attitudes of African American patients as they relate to HT | Diagnosed with HT |
| Cranney2, 2001  HCP | UK | Practice-based educational visits | To identify what is impeding GPs from implementing evidence-based guidelines in management of HT in the elderly (for an RCT) | GPs from nine practices in Merseyside |
| Crosson, 2010  HCP | USA | Outpatient primary care clinics | To assess how primary care physicians caring for patients with diabetes perceive barriers to achieving good BP control | Primary care physicians |
| Hernandez, 2012  HCP | USA | Urban, rural and suburban primary care clinics | To characterize the meaning nurse practitioners ascribed to provider/patient experiences and the NP role in health promotion and disease prevention of prehypertension | NPs caring for pre hypertensive patients |
| Howes, 2010  HCP | Australia | Southern Division of General Practice | Identify and explore barriers to initiating medication and treating elevated BP to target levels in the general practice setting | GPs and registrars |
| Howes 2, 2012  HCP | Australia | Southern Division of General Practice | Identify strategies to improve the management of hypertension in general practice | GPS |
| Hysong, 2012  HCP | USA | Veterans Affairs hospital outpatient clinics | To qualitatively identify participants’ planning and improvement strategies in hypertension care | Primary care physicians |
| Kasje, 2002  HCP | Netherlands | 1 Non-teaching and 2 university hospitals | To identify factors that may hinder or facilitate specialists use of joint treatment guidelines for primary and secondary care | Specialists |
| Parker, 2012  HCP | South Africa | Primary health care facilities | Barriers preventing the optimal utilization of health promotion | Physicians, nurses, and health educators |
| Kusuma, 2010  HCP & patients | India | Community dwellers | Perceptions of socio- economically disadvantaged migrants in Delhi regarding treatment seeking behavior for HT | Key informants, migrants from rural areas |
| Arrieta, 2009  HCP & patients | USA | Local organizations | Elicit challenges and solutions in the provision of health care to those with chronic diseases after Hurricane Katrina | Key informants, chronic diseases patients |
| HT= Hypertension  BP= Blood pressure | | | | |

| Table S3: detailed study characteristics of included quantitative studies | | | | | |
| --- | --- | --- | --- | --- | --- |
| Study, year,  Participants | **Country** | | | **Study aim** | **Population** |
| Al-Ali, 2012  HCP | Egypt | | | To understand family physicians’ reasons for not implementing WHO/ISH guidelines. | Family physicians |
| Cornuz, 2000  HCP | Switzerland | | | Importance of identified barriers to preventative interventions | General physicians |
| Flynn, 2012  HCP | Ireland | | | To quantify the use of clinical guidelines for hypertension and identify the role of ABPM in General Practice and barriers to its use | General practitioners |
| Henegan, 2007  HCP | UK | | | GPs’ awareness of current HT guidelines and their self-reported implementation of them in clinical practice | General practitioners |
| Holland, 2008  HCP | USA | | | Role of clinical inertia in the treatment of patients with HT was assessed | Physicians and support staff |
| Lin, 2006  HCP | USA | | | Factors contributing to low adherence of clinical guidelines based on clinician feedback on recommendations displayed at the point of care | Physicians and nurses caring for HT patients |
| Mahabir, 1997  HCP | Trinidad | | | Providers’ views and barriers to their practice of measuring BP, deciding the need for treatment, and selecting therapeutic drugs | Medical practitioners |
| Oliveria1, 2002  HCP | USA | | | Identify barriers to primary care physicians’ willingness to increase the intensity of treatment among patients with uncontrolled HT | Physicians treating patients with uncontrolled BP |
| Reiner, 2010  HCP | Croatia | | | To examine physicians’ knowledge and perception of CVD risk factors and barriers to guideline implementation | GPs and specialists |
| Roumie, 2007  HCP | | | USA | Provider responses to computer alerts regarding guideline recommendations for patients with suboptimal HT care | Physicians, nurses, and physician assistants |
| Schmieder, 2012  HCP | | Europe | | To understand attitude of physicians towards clinical guidelines for CVD prevention, cardiovascular risk assessment tools, and patient management in Europe | Primary care physicians, cardiologists, endocrinologists, diabetes specialists, and internal medicine specialists |
| Wang, 2004  HCP | | China | | Investigate the levels of understanding and implementation of current HT guidelines | Cardiologists |
| Waxler1, 2004  HCP | | USA | | Patient and physician barriers to HT treatment and physician decision making in the management of HT | family medicine and internal medicine physicians |
| Coleman, 2000  Patients &HCP | | USA | | Case study reemphasizes the importance of a holistic, integrated approach to any continuous quality improvement | Diagnosed with HT and uncontrolled BP |
| Dean, 2007  Patients &HCP | | UK | | To examine potential barriers to adequate BP control in patients with poorly controlled HT | Uncontrolled HT patients and their physicians |
| Kobalava, 2007  Patients &HCP | | Russia | | Problems of physician-patient cooperation and physician related barriers to target BP achievement | Diagnosed with NTH |
| Mendis, 2004  Patients &HCP | | Nigeria | | Capacity of health-care facilities in a low-resource setting to implement the absolute risk approach assess CVD risk among HT patients | Patients diagnosed with HT, Physicians, Non-physician health-care providers |
| Ahluwalia, 1997  Patients | | USA | | To identify correlates of controlled HT in a largely minority population of treated hypertensive patients | Inner city HT patients who previously filled an HT prescription |
| Bovet, 2008  Patients | | Switzerland | | To determine proportion of persons who utilized health services after being diagnosed as hypertensive | Population survey participants who had raised BP but untreated for HT |
| Cummings, 1982  Patients | | USA | | Examines the relationship of health beliefs, knowledge, and barriers to receiving care and drug treatment maintenance | HT patients previously or currently on HT treatment |
| Dennison, 2007  Patients | | South Africa | | To examine determinants of HT care and control among peri-urban hypertensive Black South Africans | Patients diagnosed HT, attended a clinic within previous 12 months |
| Edelman, 2008  Patients | | USA | | To assess follow-up practices among individuals found to have elevated cardiovascular disease (CVD) risk factors | HT, pre HT, sub optimal lipids, or abnormal blood glucose participants of the (FIT Heart) RCT |
| Gee 1, 2012  Patients | | Canada | | Self-reported adherence to antihypertensive medications, and reasons for not using and occasionally missing doses of antihypertensive drugs. | Self-reported HT patients from 2008 Canadian Community Health Survey &2009 Survey on Living with Chronic Diseases in Canada |
| Gee 2, 2012  Patients | | Canada | | Prevalence of Canadian adults with HT who use lifestyle changes to control blood pressure. And barriers to self-managing elevated blood pressure | Self-reported HT patients from the 2008 Canadian Community Health Survey |
| Gregoire, 2002  Patients | | Canada | | Examine the effects of potential predisposing, enabling and reinforcing factors on the discontinuation of initial HT medication | HT patients newly prescribed an antihypertensive monotherapy |
| Hassan, 2006  Patients | | Malaysia | | To identify the predictors of medication noncompliance among HT patients | HT patients who had been on  treatment for at least 3 months |
| Hill, 1999  Patients | | USA | | examining barriers to being in care and having adequate control of BP among African American men | HT patient participating in an RCT |
| Hong, 1006  Patients | | USA | | Barriers to adherence to anti-hypertensive medication | USA veterans diagnosed with HT |
| Hsu, 2010  Patients | | USA | | HT medication adherence in relation to the demographic attributes and the perception of need, effectiveness and safety. | Chinese American elders diagnosed with HT |
| Joyner-Grantham, 2009- Patients | | USA | | Assessed and identified gaps related to “patient inertia” factors and the control of BP | Hypertensive emergency department (ED) patients |
| Krousel-woods, 2008  Patients | | USA | | Examining barriers in post disaster situations which may reduce adherence | hypertensive patients receiving care at a multispecialty group practice |
| Mochari, 2007  Patients | | USA | | Assess BP and cholesterol knowledge, awareness of CVD risk, and factors associated with non-adherence to CVD medications and lifestyle goals. | racial/ ethnic minorities visiting Ambulatory Care in Harlem |
| Nelson2, 1978  Patients | | USA | | Examined the relationships between patients' perceptions of health, disease, medical treatment and medication compliance | HT patients on treatment |
| Oliveria2, 2005  Patients | | USA | | To assess HT knowledge, awareness, and attitudes related to SBP | HT patients from a primary care setting |
| Peltzer, 2004  Patients | | South Africa | | Examine the relationships between health beliefs variables and the use of both HT medications and alternative healing agents | HT patients attending an out-patient clinic in rural South Africa |
| Serour, 2007  Patients | | Kuwait | | To measure adherence and barriers of complying with lifestyle recommendations among patients with high cardiovascular risk factors | HT or type 2 diabetes patients, diagnosed for at least 1 year |
| Shulman, 1986  Patients | | USA | | Associations between education, socioeconomic class and economic barriers to HT medication adherence | Population survey; analysis included those with raised BP only |
| Thomas, 2011  Patients | | India | | To assess medication adherence in hypertensive patients and to identify the main barriers associated with medication adherence | HT patients treated for at least 6 months |
| Turner, 2009  Patients | | USA | | To examine the effect of antihypertensive adherence on BP and barriers to adherence in racially diverse elderly patients | HT patients with prescribed medication |
| Thrope, 2006  Patients | | USA | | To measure the association between psychological distress and adherence to USPSTF-recommended preventive care services among older adults in the | Community-dwelling elderly |
| Vawter, 2008  Patients | | USA | | To characterize the reasons for antihypertensive medication non adherence | Healthy Styles survey respondents who received prescriptions for HT medications |
| Wee, 2012  Patients | | Singapore | | To determine hypertension awareness, treatment and control, in a multi-ethnic urban lower and higher SES Asian communities in same geographic location. | General population of 2 communities with different SES status |
| Williams, 1998  Patients | | USA | | Relationship between functional health literacy level of patients and knowledge of their chronic disease and treatment | Patients with HT or diabetes presenting to general medicine clinics |
| Youssef, 2002  Patients | | Egypt | | Impediments to pharmacological and non-pharmacological compliance among patients with HT | HT patients attending health insurance clinics for prescription refills |
| HT= Hypertension  BP= Blood pressure | |  | |  |  |

| Table S4: quality appraisal of qualitative studies | | | | | | | | | | |
| --- | --- | --- | --- | --- | --- | --- | --- | --- | --- | --- |
|  | Question/ objective sufficiently described? | Study design evident and appropriate? | Context for the study clear? | Connection to a theoretical framework / wider body of knowledge? | Sampling strategy described, relevant and justified? | Data collection methods clearly described and systematic? | Data analysis clearly described & systematic? | Use of verification procedure(s) to establish credibility? | Conclusions supported by the results? | Reflexivity of the account? |
| Anthony, 2012  Patients | Yes | No | No | No | Yes | Yes | Yes | Yes | Yes | No |
| Aroian, 2012  Patients | Yes | No | No | Yes | Yes | Yes | Yes | Yes | No | No |
| Barnes, 2012  Patients | Yes | Yes | Yes | Yes | Yes | Yes | Yes | Yes | Yes | Yes |
| Horowitz, 2004  Patients | Yes | Yes | Yes | No | Yes | Yes | Yes | Yes | Yes | No |
| Fongwa, 2008  Patients | Yes | Yes | Yes | No | Yes | Yes | Yes | Yes | Yes | No |
| Ford, 2009  Patients | No | Yes | No | Yes | No | Yes | Yes | Yes | Yes | No |
| Greer, 2010  Patients | Yes | Yes | Yes | No | Yes | Yes | Yes | Yes | Yes | No |
| Machado, 2012  Patients | Yes | Yes | Yes | Yes | Yes | Yes | Yes | No | Yes | No |
| Murimi, 2010  Patients | Yes | Yes | Yes | No | Yes | Yes | Yes | No | Yes | No |
| Ogedegbe, 2003  Patients | Yes | Yes | Yes | Yes | Yes | Yes | Yes | No | Yes | No |
| Park, 2012  Patients | No | Yes | No | No | Yes | Yes | Yes | Yes | Yes | No |
| Peters, 2006  Patients | Yes | Yes | Yes | Yes | Yes | Yes | Yes | Yes | Yes | No |
| Pham, 1999  Patients | Yes | Yes | Yes | No | Yes | Yes | Yes | Yes | Yes | No |
| Schafheutle, 2002Patients | Yes | Yes | Yes | No | No | Yes | Yes | No | Yes | No |
| Wexler2, 2009  Patients | Yes | Yes | Yes | No | Yes | Yes | Yes | Yes | Yes | No |
| Cranney2, 2001  HCP | Yes | Yes | Yes | No | Yes | Yes | Yes | Yes | Yes | No |
| Crosson, 2010  HCP | Yes | Yes | No | No | Yes | Yes | Yes | Yes | Yes | No |
| Hernandez, 2012  HCP | Yes | Yes | Yes | Yes | Yes | Yes | Yes | Yes | Yes | No |
| Howes, 2010  HCP | Yes | Yes | No | No | Yes | Yes | Yes | No | Yes | No |
| Howes 2, 2012  HCP | Yes | No | No |  | Yes | No | Yes | Yes | No | Yes |
| Hysong, 2012  HCP | Yes | Yes | No | Yes | No | Yes | No | Yes | No | No |
| Kasje, 2002  HCP | Yes | Yes | No | No | Yes | Yes | No | Yes | Yes | No |
| Parker, 2012  HCP | Yes | Yes | Yes | No | No | No | No | No | Yes | No |
| Kusuma, 2010  HCP & patients | Yes | Yes | No | No | Yes | No | Yes | Yes | Yes | No |
| Arrieta, 2009  HCP & patients | No | Yes | No | No | Yes | No | Yes | No | Yes | No |

| Table S5: Quality appraisal of quantitative studies | | | | | |
| --- | --- | --- | --- | --- | --- |
| Study, year  population | Study sample represents population of interest on key characteristics, sufficient to limit potential bias to results | Response rate (%) | Exposure measures well defined and robust to misclassification bias? i.e used a validated tool | Outcome measures well defined and robust to misclassification bias? i.e objective and not self-reported, or validated if self-reported | Controlled for confounding (if applicable) |
| Al-Ali, 2012 | No | 73 | No | No | N/A |
| Cornuz, 2000  HCP | yes | 72 | no | yes | N/A |
| Flynn, 2012  HCP | No | 68 | No | No | No |
| Henegan, 2007  HCP | no | 50 | yes | no | N/A |
| Holland, 2008  HCP | yes | Not stated | no | yes | N/A |
| Lin, 2006  HCP | yes | Not stated | yes | yes | N/A |
| Mahabir, 1997  HCP | yes | 64 | no | yes | N/A |
| Oliveria1, 2002  HCP | yes | 86 | yes | yes | N/A |
| Reiner, 2010  HCP | yes | Not stated | no | no | N/A |
| Schmieder, 2012  HCP | Yes | Not stated | no | N/A | No |
| Wang, 2004  HCP | yes | 71 | no | yes | N/A |
| Waxler1, 2004  HCP | no | Not stated | yes | N/A | N/A |
| Dean, 2007  Patients &HCP | Yes | Not stated | No | Yes | No |
| Coleman, 2000  Patients &HCP | Yes | Not stated | No | Yes | No |
| Kobalava, 2007  Patients &HCP | Yes | 71 | No | Yes | N/A |
| Mendis, 2004  Patients &HCP | Yes | 51 | No | Yes | N/A |
| Ahluwalia, 1997  Patients | Yes | 51 | No | No | Yes |
| Bovet, 2008  Patients | Yes | 77 | No | Yes | yes |
| Cummings, 1982  Patients | No | 66 | No | Yes | no |
| Dennison, 2007  Patients | No | Not stated | Yes | No | yes |
| Edelman, 2008  Patients | Yes | Not stated | No | Yes | yes |
| Gee1, 2012  Patients | Yes | NA | No | No | Yes |
| Gee2, 2012  Patients | Yes | 78 | No | Yes | N/A |
| Gregoire, 2002  Patients | Yes | 98 | No | Yes | yes |
| Hassan, 2006  Patients | Yes | 98 | Yes | Yes | yes |
| Hill, 1999  Patients | Yes | Not stated | Yes | Yes | N/A |
| Hong, 1006  Patients | Yes | Not stated | yes | Yes | no |
| Hsu, 2010  Patients | Yes | 94 | Yes | Yes | no |
| Joyner-Grantham, 2009- Patients | Yes | 87 | Yes | No | no |
| Krousel-woods, 2008  Patients | Yes | 90 | No | No | yes |
| Mochari, 2007  Patients | Yes | Not stated | No | Yes | yes |
| Nelson2, 1978  Patients | Yes | 77 | No | No | yes |
| Oliveria2, 2005  Patients | Yes | 72 | No | No | yes |
| Peltzer, 2004  Patients | Yes | Not stated | No | No | no |
| Serour, 2007  Patients | Yes | Not stated | No | Yes | no |
| Shulman, 1986  Patients | Yes | 86 | No | Yes | N/A |
| Thomas, 2011  Patients | Yes | Not stated | Yes | No | no |
| Thrope, 2006  Patients | Yes | Not stated | Yes | No | N/A |
| Turner, 2009  Patients | Yes | 67 | Yes | No | yes |
| Vawter, 2008  Patients | No | 63 | No | No | yes |
| Wee, 2012  Patients | Yes | 78 | No | No | N/A |
| Williams, 1998  Patients | Yes | 71 | No | Yes | no |
| Youssef, 2002  Patients | Yes | Not stated | No | No | yes |

| **Table S6: Counts and examples of barriers per theme among qualitative studies** | | | |
| --- | --- | --- | --- |
|  | **Number of studies reporting each theme** | **Examples of patient barriers (n=17)^1^** | **Examples of provider barriers (n=10)^1^** |
| **capability** | **Knowledge**  N=9 patient studies  N=2 provider studies | - HT is a temporary disease (Anthony) - Service availability (Murimi) - Consequences and risk factors of HT (Pham, Kusuma, Barnes, Carol, Waxler2(Anthony) (Mochado) - BP readings and normal levels (Barnes) - The need for HT education classes (Pham) and diet programs to manage and prevent HT (Barnes) (Peters) | - Dealing with elderly comorbidities (Howes 1). - Guidance on home BP monitoring (Howes 2) |
|  | **Skills**  N=2 patient studies  N= 3 provider studies | - Patient communication skills (Ogedebe) - Checking BP at home (Barnes) | - Keeping up with new clinical information (Howes) - Training in education and counseling (Parker) - Addressing pre hypertension (Hernande) |
| **Intention** | **Motivation and goals**  N=2 patient studies  N=3 provider studies | - Too lazy and too tired to exercise (Ford) - Not putting enough effort or thought into treatment (Anthony) | - "I’m sure that at 6 o’clock on Friday, I’m not that fussed whether its 160 or 164- I just want to go home" (cranny 2) - “. . .as a caregiver you get tired of saying the same thing over and over and over again” (Parker) - Difficulties and failures in addressing health promotion and lifestyle changes (Hernande) |
|  | **Beliefs about consequences**  N=7 patient studies  N=5 provider studies | - No need for medications if no symptoms (Schaufleid, Ogedegbe, Aroian) - Dependence on medication (Fongwa, Ogedegbe) - Doubts over association between challenging lifestyle and HT (Carol, Waxler2) - Denial of hypertension diagnosis (Anthony) - Preference of lifestyle modifications over medical treatment (Aroian) - Fatalistic perspective: “It’s all in God’s hands” (Peters) or “it runs in the family there is nothing I can do about it” “(Waxler2) | - Uncertainty regarding accuracy and representativeness of individual BP readings (Howes) - Doubted the efficacy of certain medications (Kusuma) - Reluctant to initiate aggressive anti-hypertensive drug treatment due to possible side effects (Cranny2) - Following guidelines will not improve outcomes (Kajse) - Accuracy and representativeness of individual BP readings during the visit (Howes) (Howes2) |
|  | **Nature of behavior (Breaking habit)**  N=7 patient studies  N= 1 provider study | - Difficulty changing dietary habits “change is hard,” “habits are hard to break,” (Fongwa, Greer, Waxler2, Carol, Park, Peters). - Difficulty of long term medication adherence (Ford, Ogedegbe, Kusuma). | - Routine and satisfaction with current behaviour (Kasje) |
|  | **Social influence**  N=6 patient studies  N= 3 provider studies | - Lack of family support as a barrier to eating healthy food (Ford) and to clinical care (Ogedegbe)(Kusuma) (Murimi) - Having to cook for oneself differently from the rest of the family (Ford, Carol, Waxler2, Park) - Spending time with other Hispanics created fairly regular opportunities for unhealthy eating (Aroian) - Social pressure: Healthy behaviors, including exercise may not be socially acceptable (Aroian, Peters) - Being overweight viewed as preferable or “healthy” in some cultures (Aroian, Peters) | - Reluctance to initiate treatment in ‘someone else’s patient’ (Howes), - Poor coordination between different practices (Crosson) (Hernande) (Howes2) - Achieving consensus in practice ‘Standardization of measurement (Howes2) |
|  | **Behavioral regulation (Priority setting)**  N= 8 patient studies  N= 2 provider studies | - Work schedule and family obligations interfered with exercise, diet, attending clinic visits and overall HT control HT (Murimi, Schafelid, Greer, Waxler2, Ford, Aroian, Park) - Changing diet, quitting smoking and exercise disrupts lifestyle (Anthony) | - Acute medical conditions compete with HT during the visit (Crosson, Kasje) |
|  | **Professional identity/ agreement with guidelines**  N= 3 provider studies | NA | - Lack of trust in guidelines (Howes) (Kajse) - Impracticality of guidelines (Cranny2) - Ambiguous role identity, and nurses not differentiated from physician assistants (Herande) |
|  | **Beliefs about capabilities**  N=1 provider study | NA | - Beliefs that providers cannot perform according to the guidelines (Kasje) |
|  | **Emotion (Stress and anxiety)**  N= 4 patient studies | - Stress is a major cause to non-adherence and lack of HT control (Carol, Fangwa, Ford, Waxler2, Anthony) | NA |
|  | **Memory and attention**  N= 4 patient studies | - Forgetting to take medication (Greer) (Ford) (Ogedegbe) (Barnes) | NA |
| **Healthcare system** | **Availability**  N= 13 patient studies  N= 9 provider studies | - Lack of exercise facilities (Ford, Peters), grocery stores with healthy foods (Fongwa), dietary counselling (Carol), and health care facilities (Kusuma, Peters) - Limited food choices when eating out (Park) - Transportation difficulties affecting clinic visits (Pham, Barnes) and medication refills (Greer, Barnes) - Timing of screening services conflict with working ours (Murimie) - Difficulties getting clinic appointments (Ogedegbe) - Short duration of consultation time (Kusuma) - Lack of interpreter services (Pham) and information targeted specifically to African American health (Waxler2). - Commercials only encourage bad eating habits (Peters) | - Lack of consultation times (Cranny2, Crosson, Kasje, Kusuma, Parker, Herande) - Lack of space and equipment and shortage in staff (Parker) - Disruption of treatment due to severed supply channels and inoperable pharmacies after disasters (Martha). - Difficulties in locating guidance to providing care (Howes) - Guidelines are available but inaccessible (Parker) - Need simpler guidelines that are practical at the general practice (Howes 2) |
|  | **Affordability/ financing**  N= 9 patient studies  N= 5 provider studies | - lack of insurance, out of pocket payments, and high costs of treatment (Shawfild, Aroian, Barnes), resulting in seeking care only in acute problems (Pham, Ford, Greer, Waxler2, Martha, Kusuma) - Healthy food (Waxler2, Ford, Carol, Aroian, Peters) and exercise facilities (Peters) are too costly | - Insufficient financial reimbursement and incentives (Crosson, Kasje, Cranny2, Hernande, Howes2) |
|  | **Acceptability**  N= 6 patient studies | - Lack of respect (Kusuma), lack of attention (Barnes), and unfair treatment (Greer). - Provider- patient communications (Ogedegbe) - Lack of trust in the services provided (Greer, Peters) | NA |
|  | **Medication Related**  N= 5 patient studies | - Medication side effects (Schawfil, Fangwa, Ford, Ogdegbe, Anthony ) - Dosing frequency, taste, and large pill size (Ogedegbe). | NA |
| 1 The two studies with providers and patient barriers are included in both columns - HT = hypertension- NA= not available | | | |
